# An evaluation of gonadotropin-releasing hormone analogue administered to gilts and sows on subsequent reproductive performance and piglet birth weight

**DOI:** 10.1186/s40813-016-0018-1

**Published:** 2016-01-11

**Authors:** Frédéric Vangroenweghe, Lieve Goossens, Jan Jourquin

**Affiliations:** Elanco Animal Health, Plantijn en Moretuslei 1A (3rd floor), B-2018 Antwerpen, Belgium

**Keywords:** Peforelin, GnRH analogue, Farrowing efficiency index, Live piglet index, Reproduction, Piglet birth weight

## Abstract

**Background:**

The present study investigated the effect of peforelin (Maprelin®), a gonadotropin-releasing hormone (GnRH) analogue, administration in gilts, primiparous and pluriparous sows in a high productive farm on sow reproductive performance and piglet quality at birth.

**Methods:**

In a 400 sow herd, gilts, primiparous and pluriparous sows were randomly allocated to 2 groups: peforelin treated (peforelin = P-group) or no treatment (control = C-group). Animals were injected 48 h after the last altrenogest treatment (gilts) or 24 h post weaning (sows). Weaning-to-estrus interval (WEI), estrus rate (ER), farrowing efficiency index (FEI), farrowing rate (FR), number of total (TBP), live (LBP) and stillborn piglets (SBP), mummies (MM) and live piglet index (LPI) were assessed and compared between treatment groups. To assess piglet quality at birth, 6033 piglets from 426 litters were weighed individually within 24 h after birth (BW; birth weight).

**Results:**

No significant difference between treatment groups could be observed for WEI, TBP, LBP, SBP and MM. The ER was significantly (*P* = 0.0119) higher (93.2 %) in the P-group as compared to the C-group (87.2 %). Peforelin treatment did not affect farrowing rate. Both FEI and LPI were significantly (*P =* 0.0078) better in the P-group as compared to the C-group. Overall, no effect of peforelin treatment on piglet birth weight could be observed, although specific subcategories (1st parity and older (5+ parity) sows) did have a significant impact of treatment on birth weight. During late summer (August-September) all treated gilts and sows took advantage from peforelin treatment with a significant improvement of piglet birth weight.

**Conclusion:**

Peforelin treatment had a significant impact on ER, FEI and LPI. Moreover, piglet birth weight improved for specific sow subcategories (1st parity and older sows) and for all gilts and sows during the late summer infertility period.

## Background

Optimal reproductive performance is crucial for economic success in commercial pig herds [28, 32]. Different management strategies [24], such as optimized feeding strategies, hyperprolific dam lines [3], batch farrowing systems and extended photoperiod during the post-weaning phase [6], are applied in order to meet the high performance expectations of modern sow farmers [18, 34]. However, inevitable variations in farm conditions, such as season [24, 25], infection pressure or feed ingredients [10, 18] can negatively impact the results of high productive genetics. Moreover, recent evidence has shown that reproductive performance is influenced by wean-to-estrus (WEI) interval [26].

Pharmaceuticals, such as progesterone-analogues and gonadotropins, are used in practice to control reproduction with the aim to increase the reproductive performance of gilts and sows [8]. The use of gonadotropins post-weaning in sows or following altrenogest treatment in gilts, both in order to stimulate the follicular development, can achieve an even better synchronization effect [5, 20]. In the mature pig, natural gonadotropin secretion is controlled through the release of hypothalamic peptides, gonadotrophin-releasing hormones (GnRH), which pass through the hypophyseal portal vessels to the pituitary gonadotrophs, causing the release of follicle-stimulating hormone (FSH) and luteinizing hormone (LH) [22]. GnRH regulates both FSH, thus playing a key role in growth and maturation, and LH to a certain extent, with final ovulation of the follicles [5, 21].

The positive influence of peforelin, a I-GnRH-III which induces an FSH-increase and no LH-increase [38, 39], on the estrus induction in sows has been shown in Germany [13, 14]. Using peforelin, the interval between the last altrenogest treatment and estrus could be reduced [11, 12] and an effect on the depression of fertility performance during the warmer season was shown [13]. Recently, the use of peforelin in high performing farms has been shown to shorten the weaning-to-estrus interval in sows [1]. Other parameters which can be influenced by the use of peforelin is the farrowing efficiency index (FEI = litters per 100 treatments; [16]) and subsequent litter performance [16, 17]. Following peforelin treatment, an FEI of 82 % was obtained as compared to 79 % in the placebo group [16].

The purpose of the present study was to investigate the effect of peforelin treatment in gilts after altrenogest treatment and in sows post weaning on piglet quality (birth weight) at birth and sows reproductive performance in a high productive well-managed sow herd.

## Results and discussion

### Litter and reproductive performance

All results concerning litter performance (WEI, LBP, SBP and MM) and reproductive parameters (ER, FR, FEI and LPI) are shown in Table 1. Taking into account only the gilts and sows in estrus until 7 d pw (*n* = 484), no significant difference in WEI could be detected between both treatment groups. In the present study, only gilts and sows in estrus until 7 d pw were taken into account, since it has been shown that sows coming into estrus later than 7 d pw are subfertile and from 9 d pw onwards, a different surge of ovarian follicles is involved, which implies that comparison of post-weaning estrus results becomes impossible [30]. All parameters concerning number of piglets born were not significantly different. The ER showed significant (*P* < 0.05) improvement following peforelin treatment (P-group) as compared to the control. Treatment with peforelin did not impact (*P* < 0.05) the FR. The FEI was significantly (*P* < 0.05) better in the P-group (79.2 %) as compared to the C-group (70.2 %), resulting in a significant effect on the LPI (C-group, LPI = 1032; P-group, LPI = 1151; *P* < 0.01).

**Table 1:** Reproductive parameters (WEI; LBP, live born piglets; SBP, stillborn piglets; MM, mummies) and reproduction indicators (ER, estrus rate; FR, farrowing rate; FEI, farrowing efficiency index; LPI, live piglet index) for both treatment groups (C-group, control group, *n* = 289; P-group, peforelin-treated group, *n* = 279) and different parity groups (gilts, 1st parity sows, 2^nd^–4^th^ parity sows and 5+ parity sows), expressed as means ± SEM and their significance level

	C-group	P-group	Significance level
WEI overall	6.11 ± 0.33	6.82 ± 0.44	*P* > 0.05
WEI ≤ 7 d pw	5.08 ± 0.26	5.10 ± 0.34	*P* > 0.05
LBP	14.7 ± 0.20	14.4 ± 0.19	*P* > 0.05
LBP – gilts	13.76 ± 0.49	13.95 ± 0.47	*P* > 0.05
LBP – 1^st^ parity	14.47 ± 0.61	13.39 ± 0.53	*P* > 0.05
LBP – 2^nd^–4^th^ parity	15.07 ± 0.22	14.73 ± 0.23	*P* > 0.05
LBP – 5+ parity	13.40 ± 0.079	13.83 ± 0.54	*P* > 0.05
SBP	0.18 ± 0.07	0.80 ± 0.08	*P* > 0.05
MM	0.38 ± 0.07	0.28 ± 0.04	*P* > 0.05
ER	87.2 %	93.2 %	*P* < 0.05
FR	81.3 %	85.7 %	*P* > 0.05
FEI	70.9 %	79.9 %	*P* < 0.05
FEI – gilts	42.9 %	82.6 %	*P* < 0.0001
FEI – 1^st^ parity	65.5 %	74.2 %	*P* > 0.05
FEI – 2^nd^–4^th^ parity	78.5 %	82.5 %	*P* > 0.05
FEI – 5+ parity	71.4 %	68.5 %	*P* > 0.05
LPI	1032	1151	P < 0.01
LPI – gilts	590	1152	P < 0.0001
LPI – 1^st^ parity	948	994	P > 0.05
LPI – 2^nd^–4^th^ parity	1183	1215	*P* > 0.05
LPI – 5+ parity	957	947	*P* > 0.05

The present study investigated whether peforelin, a synthetic I-GnRH-III, could influence litter performance, reproductive parameters and piglet birth weight in gilts and sows in a high productive sow herd during different seasonal reproductive conditions.

Although numerical difference could be observed in litter performance (WEI, LBP, SBP and MM), the treatment with peforelin did not show a significant impact on any of these parameters. Between both treatment groups, the WEI showed no significant decrease in the treatment group as compared to the control group. These results are in accordance with Engl et al. [14]. In these studies no differences were observed in WEI between treatment groups, except for the primiparous sows treated with PMSG, a product which was not included in the present study.

The FEI, takes into account the effect of the treatment on ER and the number of inseminated sows that farrow as a result of the insemination following treatment. In this way, it differs from the traditional FR. Furthermore, the traditional FR is based on the total number of sows inseminated and thus also taking into account the litters of sows returned into estrus. Since the present study compares peforelin treatment, a product to induce estrus, with an untreated control, the innovative performance parameter FEI is more appropriate to benchmark the reproductive results between both treatment groups. The 8.95 % higher FEI in the treated group compared to the control group (Table 1) can be mainly attributed to a significant difference in FEI in the gilt subcategory, although the 1st and 2nd-3rd parity sows also showed a non-significant, numerical tendency for improvement. No significant difference in FEI throughout the different study periods could be observed, although numerical trends showed an improvement in all study periods. This indicates that GnRH-agonists have little influence on the outcome of pregnancy [19, 23] and mainly improve estrus performance. An increase of almost 9 % in FEI may have a significant biological impact on sow farm management, especially under conditions of batch management systems, where return-to-estrus and non-pregnancy can have a major impact on the batch stability. Looking at FEI in further detail, the impact of peforelin treatment doubled (*P* < 0.05) FEI (45 % in C-group to 79 % in P-group) in gilts treated after altrenogest synchronization.

The results obtained in the present study were from a farm with good reproductive results (32.4 piglets/sow/year), although the reproductive results of the gilts in the control group could clearly be identified as problematic. However, a previous study [9] performed in farms with more average reproductive results (approx. 26 piglets/sow/year) also showed significant effects on estrus rate. Therefore, it can be concluded that peforelin treatment can be implemented on swine farms with various levels of reproductive performance.

Total born piglets (sum of LBP and SBP) did not differ between control and treatment group, indicating the safety of the product. Although the number of LBP was slightly lower in the treated group, the LPI was higher overall due to the better FEI in the treated animals.

### Birth weight

**Fig. 1:**
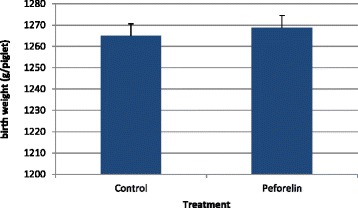
Birth weight (average ± SEM) per treatment group (C-group, control group, *n* = 2985; P-group, peforelin-treated group, *n* = 3048). Significant differences (*P* < 0.05) between both treatment groups are indicated by an asterix (*)

**Fig. 2:**
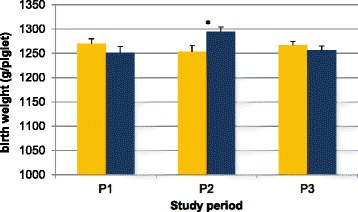
Birth weight (average ± SEM) per treatment group (C-group, control group = yellow; P-group, peforelin-treated group = blue) blocked by season (S1, June–July, LBP = 14.50 ± 0.26; S2, August–September, LBP = 13.94 ± 0.25; S3, October–November–December, LBP = 14.87 ± 0.20). Significant differences (*P* < 0.05) between both treatment groups are indicated by an asterix (*)

**Fig. 3:**
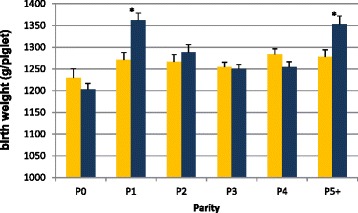
Birth weight (average ± SEM) per treatment group (C-group, control group = yellow; P-group, peforelin-treated group = blue) blocked by parity (P0, gilts, LBP = 13.88 ± 0.35; P1, 1st parity, LBP = 13.88 ± 0.40; P2–4, 2nd to 4th parity, LBP–P2 = 14.79 ± 0.39, LBP–P3 = 14.80 ± 0.24, LBP–P4 = 15.12 ± 0.26; P5+, 5th and older parities, LBP = 13.61 ± 0.48). Significant differences (*P* < 0.05) between both treatment groups are indicated by an asterix (*)

All results concerning birth weight and the effect of treatment, parity and season are shown in Figs. 1, 2 and 3. Overall, treatment with peforelin did not result in a significant increase in BW (Fig. 1). The coefficient of variation (CV) was identical (24.9 %) in both treatment groups, indicating no overall difference in homogeneity of the litters from the C-group compared to the P-group. When stratification into parities was applied, a significant impact of peforelin treatment could be observed for 1st parity sows (*P* < 0.05) and older sows (5^th^ and older parities) (*P* < 0.05). For all other parities (gilts and 2^nd^–4^th^ parity), no significant effect of peforelin treatment could be shown throughout the study. This could possibly be explained by the difference in the number of TBP among the different groups. Although these differences were non-significant, 1st parity sow and 5th and older parities had approximately 0.5 to 1 piglets less born per litter as compared to 2^nd^-4^th^ parity sows, which is could partly explain the difference of around 100 g of BW in both these age groups [3]. Another explanation could be that a uniform ovulatory follicle pool at the ovary would result in a uniform oocyte quality, and an improved the luteal development [40] resulting in a better embryo quality [27, 37]. These elements could finally result in more uniform birth weights [35]. Jourquin and Goossens [17] have also shown a positive impact of peforelin treatment on the performance of the subsequent litter, which can be explained by increase of FSH, needed for follicle development [15], through peforelin treatment, resulting in larger follicles and a better pre-ovulatory follicle pool with more competent follicles to ovulate [11]. First parity sows have a higher chance of suffering from bad reproductive performance in their next cycle [29], and therefore the effect of peforelin treatment on this animal category could have a greater impact on subsequent litter quality and reproductive parameters. Older sows on the otherhand, may suffer from lower reproductive performance, since this is the major cause of culling at higher parities [7, 9]. From a practical point of view, peforelin treatment of specific subcategories of sows within a high productive sow farms could be advised throughout the year for optimization of reproductive performance.

The effect of seasonality was significant (*P* < 0.05) with higher BW in gilts and sows treated with peforelin and inseminated during late summer (P2, August–September). In other periods (P1, June–July and P3, October–November–December), no effect of peforelin treatment on BW could be shown.

Late summer infertility and related low reproductive performance has been described [24, 25]. The effect was observed in the period of maximal summer infertility due to photoperiod effects, namely from week 28 till week 42 [2]. The seasonal change in circadian melatonin release alters hypothalamic GnRH release, subsequently affecting LH pulse amplitude and/or frequency [31]. After the first embryonic oestrogenic signal, if the supply of LH is inadequate, CL production of progesterone may be insufficient to support appropriate embryonic development [4]. Therefore, treatment with peforelin, a GnRH analogue, could solve this temporary alternation in endogenous hypothalamic GnRH release. Moreover, a recent study [9] showed a positive effect of peforelin treatment on follicle growth in gilts and sows. As previous explained, this could result in a uniform oocyte quality, and an improved the luteal development [40] resulting in a better embryo quality [27, 37] during the period of summer infertility.

## Conclusions

Peforelin treatment had a significant impact on ER, FEI and LPI. Moreover, piglet birth weight improved for specific sow subcategories (1^st^ parity and parity 5 or older sows) and for all sows during the late summer infertility period. These subcategories are the sows with the highest risk of lower piglet quality, as well as the sows inseminated during the period of late summer infertility.

## Methods

### Herd description, study animals and management practices

A high productive sow herd with 400 Topigs 20 sows was selected. The number of weaned piglets/sow/year was above 32 in the last 12 months before the enrollment of the study and per batch, 80 to 92 % of the sows showed estrus within 7 days after weaning. A detailed description of the sow herd characteristics is given in Table 2.

**Table 2:** Characteristics of the high productive sow herd

	Herd characteristic
Number of sows	400
Breed	Topigs 20
Batch management system (weeks)	4
Lactation length (weeks)	3
Piglets weaned per sow per year	32.4
Average WEI (days ± SEM)	6.5 ± 0.3
Age of gilts at first insemination (days ± SEM)	270 ± 15
Type of housing for pregnant sows (<4w of gestation)	Individual Stalls
Type of housing for pregnant sows (>4w of gestation)	Pens with groups of 18 sows
Vaccinations of gilts (weeks post arrival in quarantine)	
● Parvovirus & *Erysipelothrix rhusiopathiae*	0 – 3
●* Escherichia coli*	1 – 4
● Atrophic rhinitis	1 – 4
● Porcine Reproductive and Respiratory Syndrome virus	0 – 3
Vaccinations in sows (weeks pre/postpartum)	
● Parvovirus & *Erysipelothrix rhusiopathiae*	+2
●* Escherichia coli*	- 3
● Atrophic rhinitis	- 3
● Porcine Reproductive and Respiratory Syndrome virus	- 7

In total, 568 animals (gilts, *n* = 96; 1^st^ parity sows, *n* = 60; multiparous sows, *n* = 412) were enrolled during at least one reproductive cycle. Animals with clinical symptoms and/or reproductive disorders, such as vaginal discharge or abortions, were excluded from the study. Gilts had been synchronized with altrenogest (Regumate®; MSD Animal Health, Brussels, Belgium) for 18 days (20 mg per gilt/day) after they had shown at least one estrus. To ensure correct dosing, gilts were housed in individual stalls during altrenogest treatment. After altrenogest treatment (gilts) or weaning (sows), animals were moved to a specifically designated insemination area with individual housing and a light schedule of at least 16 h per day (150–275 Lux). Repeat breeders were not included as there is no timepoint available to determine the correct dosing moment.

Estrus stimulation started at 48 h post weaning (pw) or 72 h after the last altenogest treatment in gilts, using a teaser boar. All animals were fed *ad libitum* during the pre-estrus period. Following insemination and throughout gestation, a strict feeding schedule was applied with a lower feeding level after insemination, increasing after 40 d in gestation.

### Experimental design

The main objectives of the study were to determine the effect of the peforelin treatment on piglet quality, defined as piglet birth weight within 24 h after birth and the effect on reproductive performance of the sows. A minimum of 250 sows was required per treatment group (C-group *vs.* P-group) to be able to detect a difference of 50 g in piglet birth weight between the treatments with 95 % confidence, 80 % power and a standard deviation of 300 g (WinEpiscope 2.0; [33]). Animals were stratified according to parity (gilts, 1^st^ parity sows, 2^nd^ to 4^th^ parity sows and 5^th^ and older parity sows) and randomly allocated to one of both treatment groups at the moment of injection: peforelin (Maprelin®) or P-group (150 μg peforelin in gilts and pluriparous sows, 37.5 μg in primiparous sows, 2 ml resp. 0.5 ml peforelin according to the manufacturers’ instructions) and negative control or C-group (no injection). Animals were injected intramuscularly on the right-hand side of the neck 24 (±2) h pw (sows) and 48 (±2) h after the last altrenogest treatment (gilts) (Table 3). The treatments were performed by the farm veterinarian following randomization by the investigator. All personnel involved in estrus detection, insemination, birth weight measurement and collection of reproductive data were blinded for the group allocation of the animals. The treatments were performed from June 2012 to January 2013.

**Table 3:** Treatment schedule for peforelin administration in different sow groups

Sow category	Peforelin dose (75 μg/ml)	Administration timepoint
Gilts	2.0 ml	48 h after last altrenogest administration
1^st^ parity sows	0.5 ml	24 h post weaning
≥2^nd^ parity sows	2.0 ml	24 h post weaning

### Study period description

To study the effects of peforelin on sows during the period with highest probability of fertility problems, the treatment period was clustered into 3 specific study periods, based on the current knowledge of seasonal reproductive problems [24, 25]. Period 1 was defined as early summer with treatment performed during June–July (P1, period 1), period 2 was defined as late summer and associated with fertility problems mainly during August–September (P2, period 2) and period 3 was defined as autumn with treatment performed during October–November–December (P3, period 3).

### Estrus detection and insemination protocol

Estrus detection was performed in sows from 48 h pw and in gilts from 72 h after the last altrenogest treatment onwards twice daily, in the morning at 9 am and in the afternoon at 16 pm, using active boar contact. Sows in estrus showing a standing reflex in the morning were inseminated in the afternoon at 17 pm, whereas sows in estrus in the afternoon were first inseminated the next morning at 10 am. If sows were still showing estrus the next day, they were inseminated 24 h after the first insemination.

### Parameters of comparison

#### Reproductive performance parameters

The estrus rate (ER) is defined as the proportion of animals that were in estrus per 100 animals included. All animals in estrus were inseminated. The farrowing rate (FR) is defined as the proportion of animals that farrowed per 100 inseminations. The farrowing efficiency index (FEI) is defined as the proportion of animals that farrowed per 100 animals included and is therefore an innovative parameter taking into account both ER and FR in only one figure. It is calculated as the number of farrowings divided by the number of gilts and sows enrolled post-weaning or post-synchronization. Only animals inseminated within 7 d pw (*n* = 484) are taken into account, since animals coming into estrus later than 7 d pw (*n* = 26) are considered as subfertile [30].

#### Litter parameters

From each litter, the number of live born piglets (LBP), stillborn piglets (SBP) and mummified piglets (MM) was recorded. Due to the study blinding, cross fostering could not be limited within a specific treatment group. Therefore, the number of weaned piglets could not be correctly allocated to treatment groups. Based on LBP and FEI, the live piglet index (LPI) was calculated. The LPI gives the number of piglets born per 100 animals included. It is calculated as sum of LBP per 100 animals that started their reproductive cycle pw or post-synchronization. This index takes into account one step more than the previously defined piglets produced per mated female (PPMF) [36].

#### Birth weight

Live born piglets were individually weighed (birth weight, BW; expressed in g) within 24 h after birth and before cross fostering.

### Statistical analysis

Normal distribution of the data was tested using Normal Quantile Plot. Values in the groups were usually expressed as means and standard error of the mean (SEM). To detect differences between groups for LPB, SBP, MM, ER, WEI, FR, FEI, LPI and BW cross tabulation and the Chi square test were used. Multiple comparison for the parameter BW was performed using ANOVA and pair wise comparisons between groups were made with the *post hoc* Bonferroni test. Significance was obtained when *P <* 0.05. The statistical calculations were performed using the software program JMP 9.0.3. (SAS Institute Inc., Cary, North Carolina, USA).

### Ethical and welfare statement

The study has been approved by the internal Lilly/Elanco approval system for post-approval studies under the number PEFORELIN-2012-BE-1. Since the study was performed according to the product label and without additional animal interventions, no need for additional ethical or welfare approval was necessary.

## Abbreviations

BW: Birth weight
ER: Estrus rate
FEI: Farrowing efficiency index
FR: Farrowing rate
FSH: Follicle stimulating hormone
GnRH: Gonadotropin-releasing hormone
LBP: Live born piglets
LH: Luteinizing hormone
LPI: Live piglet index
MM: Mummies
SBP: Stillborn piglets
TBP: Total born piglets
WEI: Weaning-to-estrus interval

## References

[1] Arnauts J, Jourquin J, Goossens L (2010). Improving oestrus behavior with peforelin, a specific FSH releasing GnRH.

[2] Auvigne V, Leneveu P, Jehannin C, Peltoniemi O, Sallé E (2010). Seasonal infertility in sows: a five year field study to analyze the relative roles of heat stress and photoperiod. Theriogenol.

[3] Beaulieu AD, Aalhus JL, Williams NH, Patience JF (2010). Impact of piglet birth weight, birth order, and litter size on subsequent growth performance, carcass quality, muscle composition, and eating quality of pork. J Anim Sci.

[4] Bertoldo KP, Schneider F, Tuchscherer A, Kanitz W (2012). Seasonal variation in the ovarian function of sows. Reprod Fertil Developm.

[5] Brüssow KP, Schneider F, Tuchscherer A, Kanitz W (2010). Influence of synthetic lamprey GnRH-III on gonadotropin release and steroid hormone levels in gilts. Theriogenol.

[6] Claus R, Weiler U (1985). Influence of light and photoperiodicity on pig prolificacy. J Reprod Fertil Suppl.

[7] Dial GD, Marsh WE, Polson DD, Vaillancourt JP, Leman AD, Straw BE, Mengeling WL, D’Allaire S, Taylor DJ (1992). Reproductive failure: differential diagnosis. Diseases of swine.

[8] de Jong E (2014). Weaning practices and culling policy: critical steps for optimal reproductive performance of female breeding pigs.

[9] de Jong E, Kauffold J, Engl S, Jourquin J, Maes D (2013). Effect of a GnRH analogue (Maprelin®) on the reproductive performance of gilts and sows. Theriogenol.

[10] Einarsson S, Rojkittikhun T (1993). Effects of nutrition on pregnant and lactating sows. J Reprod Fertil Suppl.

[11] Engl S. Untersuchungen zur Eignung einer neuen GnRH-Variante zur Brunstinduktion bei pluripare Sauen. Inaugural-Dissertation zur Erlangung des Grades eines Doctor medicinae veterinariae. Veterinärmedizinische Fakultät, Universität Leipzig, Germany. 2006.

[12] Engl S, Bischoff R, Zaremba W (2010). Use of a new GnRH to control reproduction in gilts.

[13] Engl S, Zepperitz H, Rath R, Zaremba W (2010). Practical experience with Peforelin in a large sow herd: data from primiparous sows.

[14] Engl S, Zepperitz H, Rath R, Zaremba W (2010). Practical experience with Peforelin in a large sow herd: data from pluriparous sows.

[15] Guthrie HD, Garrett WM. Apoptosis during folliculogenesis in pigs. Reprod. Suppl. 2001;58:17-29. 11980188

[16] Jourquin J, Goossens L (2011). Oestrus induction with Peforelin, new insights from the field.

[17] Jourquin J, Goossens L (2011). Impact of oestrus induction with Peforelin on subsequent litter performance.

[18] Kim SW, Weaver AC, Shen YB, Zhao Y (2013). Improving efficiency of sow productivity: nutrition and health. J Anim Sci Biotechnol.

[19] Manjarin R, Garcia JC, Dominguez JC, Castro MJ, Alegre B, Munoz JD, Kirkwood RN (2010). Effect of gonadotropin treatment on estus, ovulation and litter size in weaned and anestrus sows. J Anim Sci.

[20] Martinat-Botté F, Venturi E, Guillouet P, Driancourt MA, Terqui M (2010). Induction and synchronization of ovulations of nulliparous and multiparous sows with an injection of gonadotropin-releasing hormone agonist (Receptal). Theriogenol.

[21] McCann SM, Karanth S, Mastronardi CA, Dees WL, Childs G, Miller B, Sower S, Yu WH (2001). Control of gonadotropin secretion by follicle-stimulating hormone-releasing factor, luteinizing hormone-releasing hormone and leptin. Arch Medic Res.

[22] McCann SM, Marubayashi U, Sun H-Q, Yu WH (1993). Control of follicle-stimulating hormone and luteinizing hormone release by hypothalamic peptides. Ann NY Acad Sci.

[23] Patterson JL, Willis HJ, Kirkwood RN, Foxcroft GR (2010). Lack of an effect of prostaglandin injection at estrus onset on the time of ovulation and on reproductive performance in weaned sows. Theriogenol.

[24] Peltoniemi OA, Love RJ, Heinonen M, Tuovinen V, Saloniemi H (1999). Seasonal and management effects on fertility of the sow: a descriptive study. Anim Reprod Sci.

[25] Peltoniemi OAT, Virolainen JV (2006). Seasonality of reproduction in gilts and sows. Soc Reprod Fertil.

[26] Poleze E, Bernardi ML, Amaral Filha WS, Wentz I, Bortolozzo FP (2006). Consequences of variation in weaning-to-estrus interval on reproductive performance of swine females. Livestock Sci.

[27] Pope WF, Xie S, Broermann DM, Nephew KP (1990). Causes and consequences of early embryonic diversity in pigs. J Reprod Fertil Suppl.

[28] Smith C, Dickerson GE, Tess MW, Benett GL (1983). Expected relative responses to selection for alternative measures of life cycle economic efficiency of pork production. J Anim Sci.

[29] Soede NM, Hoving LL, van Leeuwen JJL, Kemp B (2012). Suboptimal reproductive performance of second parity sows: causes, consequences and use of post-weaning altrenogest.

[30] Steverink DWB, Soede NM, Groenland GJR, van Schie FW, Noordhuizen JPTM, Kemp B (1999). Duration of estrus in relation to reproduction results in pigs on commercial farms. J Anim Sci.

[31] Tast A, Peltoniemi O, Virolainen JV, Love RJ (2002). Early disruption of pregnancy as a manifestation of seasonal infertility in pigs. Anim Reprod Sci.

[32] Tess MW, Benett GL, Dickerson G (1983). Simulation of genetic changes in life cycle efficiency of pork production. II. Effects of components on efficiency. J Anim Sci.

[33] Thrusfield M, Ortega C, Dde-Blas I, Noordhuizen JP, Frankena K (2001). WIN EPISCOPE 2.0: improved epidemiological software for veterinary medicine. Vet Rec.

[34] Vangroenweghe F, Suls L, Van Driessche E, Maes D, De Graef E (2012). Health advantages of transition to batch management system in farrow-to-finish pig herds. Czech Vet J.

[35] Wientjes JGM, Soede NM, Van den Brand H, Kemp B (2012). Nutritionally induced relationships between insulin levels during the weaning-to-ovulation interval and reproductive characteristics in multiparous sows: II. Luteal development, progesterone and conceptus development and uniformity. Reprod Domest Anim.

[36] Wilson MR, Dewey CE (1993). The associations between weaning-to-estrus interval and sow efficiency. Swine Health Prod.

[37] Xie S, Broermann DM, Nephew KP, Geisert RD, Pope WF (1990). Ovulation and early embryogenesis in swine. Biol Reprod.

[38] Yu WH, Karanth S, Sower SA, Parlow AF, McCann SM (2000). The similarity of FSH-releasing factor to lamprey gonadotropin-releasing hormone III (l-GnRH-III). Proc Soc Experiment Biol Med (New York).

[39] Yu WH, Karanth S, Walczewska A, Sower SA, McCann SM (1997). A hypothalamic follicle-stimulating hormone-releasing decapeptide in the rat. Proc Natl Acad Sci U S A.

[40] Zak LJ, Xu X, Hardin RT, Foxcroft GR (1997). Impact of different patterns of feed intake during lactation in the primiparous sows on follicular development and oocyte maturation. J Reprod Fertil.

